# A push for public health: the effect of e-bikes on physical activity levels

**DOI:** 10.1186/s12889-017-4817-3

**Published:** 2017-10-16

**Authors:** Hanne Beate Sundfør, Aslak Fyhri

**Affiliations:** Institute of Transport Economics, Gaustadalleén 21, 0349 Oslo, Norway

**Keywords:** E-bike, Physical activity, Substitution effect, Public health

## Abstract

**Background:**

Cycling is considered to have a positive effect on public health through increased physical activity. In Norway, the e-bike is seen as a way of getting more people to cycle. However, the motorized assistance of an e-bike potentially eliminates any physical activity associated with its use. It is possible that the assumed health effect of increased cycling is “erased” through a reduction in other physical activities (a substitution effect). In this paper we study the public health effects of e-bikes using a combined cross-sectional and quasi-experimental design. First, we explore the existence of potentially hedonistic values in relation to interest in acquiring an e-bike and, second, we conduct an intervention study of physical activity pre- and post-purchase.

**Methods:**

A sample of 340 people responded to a questionnaire before buying an e-bike and follow-up 4 weeks later, when 45 had bought one. A further 28 (mainly physically inactive) were recruited through a Norwegian NGO. For a comparison group, 1995 people were recruited through the Falck National Register of Bicycle Owners. All respondents were asked about the intensity of their cycling, (kilometres cycled in the previous week), walking and physical activity in addition to cycling as means of transport (days and hours).

**Results:**

A structural equation model showed that hedonistic life values, and general physical activity, were predictive of interest in buying an e-bike. However people who already cycled a lot showed less interest. The trial showed that increased cycling – whether as a mean of transport or exercise –was related to higher levels of total physical activity in both groups compared to a comparison group (one-way ANOVA).

**Conclusions:**

Our findings indicate that in the Norwegian cycle population there is no substantial substitution effect of physical activity with the introduction of an e-bike. The appeal of the e-bike is strongest among those with little existing interest in, or levels of, physical activity. The net effect of the e-bike therefore seems positive from a public health perspective.

**Electronic supplementary material:**

The online version of this article (10.1186/s12889-017-4817-3) contains supplementary material, which is available to authorized users.

## Background

In Norway, the annual cycling share is approximately 5 % (with a seasonal variation from one to 8%) [23]. Increasing cycling as a mode of transport is a political goal [3]. In part due to the potential for increased overall physical activity and reduced sedentarism. There is strong evidence for the positive health effects of physical activity, in terms of overall reduced mortality and potential avoidance, or delay, in outbreaks of lifestyle-induced disease (e.g. cardiovascular disease, stroke, colon cancer, breast cancer and type II diabetes) [9]. The health benefits of physical activity (PA) depends on an individual’s baseline fitness (i.e. weight, maximal O_2_ uptake) and the frequency, duration and intensity of the activity performed [4, 17]. The Norwegian Directorate of Health recommends that adults engage in at least 150 min of moderate-intensity physical activity per week [41], accumulated through activity bouts of at least 10 min [47]. The intensity of physical activity is indicated by the metabolic equivalent of the task (MET),^1^ with the minimum goal in the range 500–1000 MET min per week [21]. Although vigorous physical activity can produce highest level of fitness, the greatest improvements in health are obtained by people who progress from sedentary to moderately active [46]. This means that, a moderate level of activity is most relevant from a public health point of view.

Previous studies have found that substantial physical activity can be accumulated through active travel [10, 34]. E.g. cycling at >15 km/h on a regular bike induces sufficient effort to adequately stimulate fitness [38] and is categorized as moderate activity (MET value of 4) [1]. In line with these estimations, someone changing from a passive transport mode (i.g. car) to bicycle for a total round-trip distance of 7.5 km a day (about 30 min overall, 15 min per trip) would meet the minimum recommendation for physical activity. Hence, increasing the amount of active travel is key for reaching targets for improved public health. It should be noted that an important additional way in which active travel enhances health is by reducing *sedentarism*. A lifestyle characterised by prolonged time spent in uninterrupted sedentary behaviours (e.g. office-based work and TV watching) can provide health risks independently regardless of engagement in physical exercise [13, 30]. Most active travellers engage in brief trips (10–15 min) twice a day [33], providing important “breaks” in a daily routines characterized by prolonged sedentarism.

In Europe, the sales of e-bikes with an integrated battery assisting the rider’s own pedal-power are increasing [7]. According to European Union regulations, e-bikes are classified legally as bicycles if they fulfil certain criteria, including assistance up to a maximum speed of 25 km/h. In European countries such as Germany, the Netherlands and Switzerland, e-bikes now comprise between 15 and 20% of annual sales [7]. In Norway, e-bikes represent only about 1% of annual bicycle sales [7], but the numbers are increasing.

It has been suggested that e-bikes might result in less physical activity than regular bicycles [24]. However, research has indicated that even though they provide decreased physical activity compared to traditional bikes, they still achieve a level necessary for health enhancement [12, 18]. E-bikes can in fact help overcome perceived barriers to cycling, such as hills or too long distance to destination, yet making the cyclist doing a considerable effort by actively pedalling. Another important aspect is that these benefits might be appealing for certain journeys and for certain people, hence recruiting “new” cyclists. A study in the Netherlands has concluded that giving rewards for commuting by e-bike yields positive results regarding the mobility and health of commuters [43], but more studies are needed if we are to be more informed about the actual effects [12].

It has been suggested that the assumed health effect of increased cycling can be cancelled out by a compensatory reduction in other physical activities (a substitution effect). However, the empirical evidence for substitution is weak [42]. Moreover, fitness gains from increased cycling could inspire individuals to be more active in other domains, hence increasing *overall* physical activity. Previous studies in adults, would indicate that interventions targeting one specific health behaviour can have a motivational impact on others [29]. Experiencing improved physical fitness and a sense of achievement could in themselves be important factors in increasing and maintaining physical activity [39].

Previous studies in Norway, show that the e-bike is of little interest to people who already cycle a lot for transport or for exercise [16] – and that the e-bike thus serves other purposes and markets than the ordinary bike.

It has been suggested that in countries with low cycling levels, such as Norway, there is a large proportion of training-oriented and highly equipped cyclists – so-called “lycra-cyclists” [14] – and in such a cycling culture the e-bike is counter to the motivation for cycling (namely that it provides daily exercise). In support of this, a previous study has shown that there is a substantial part of the cycling population that has *improved fitness* as a main motivation for cycling [16]. However, an equally large segment of the cycling population has been found not to be motivated by fitness. Hence, fitness turns out to be the most divisive issue related to cycling: people tend to think of fitness as either important or unimportant in their decision about mode of travel, and rarely as of middle importance [16].

This disparity in motivation for cycling is a good illustration of the importance of looking not just at extrinsic motivations but also at intrinsic motivations for travel if we are to better predict travel demand [31]. Furthermore, it points to the importance of studying how people differ in their evaluation of different types of motivation. The theory of planned behaviour (TPB) is often used to predict behaviour [2]. Studies have linked socio-psychological variables derived from the TPB such as attitudes, social norms and perceived behavioural control to active transport (cycling and walking) [11, 22, 28]. In a review of psychological determinants of active travel [32] concluded that the TPB variables could predict existing levels of active transport, but often failed to predict *changes* in behaviour. A promising alternative to the TPB is to look at people’s *value systems.* Values are “desirable, trans-situational goals, varying in importance, that serve as guiding principles in people’s lives” ([37], p. 269). As opposed to *attitudes*, that are seen as more superficial and rationally derived, values are believed to be deeper engraved into a person’s sense of self, and hence to function more as sources of intrinsic motivation for behaviour ([37], p. 269). *Hedonism* is a value within Shwartz’s value system that implies priority to pleasure and sensuous gratification being given as a goal relative to other important goals [35]. People who score highly on Hedonism have been found to be less likely to conduct positive health behaviours [45]. Including information about people’s hedonistic life values within the context of physical activity could improve ability to predict choice of the e-bike as transport mode, and subsequently their potentially increased *active mobility* (use of non-motorized transport). As far as we know, no previous studies have attempted to link Hedonism to choices about active mobility. A plausible hypothesis in that regard is that people who score high on Hedonism are less likely to engage in physical activity and to travel by active mobility. Further, e-bikes might have a stronger appeal among people who score high on Hedonism.

Adopting a public health perspective, we explore perceptions related to e-bikes and physical activity in the general public, with a particular focus on how these factors relate to hedonistic life values. In this way, we aim to expand our understanding of the potential spread of e-bikes. Furthermore, we test the changes in physical activity of a given individual who obtains an e-bike compared to someone who does not. Finally, we investigate the potential substitution effect, i.e. whether the physical activity that may occur from increased cycling activity is associated with reduced physical activity in other domains.

## Method

### Sample and procedure

A convenience sample of interested in buying an e-bike were recruited through posters in bike shops (in Oslo) and through social media (i.e. Facebook, Twitter). The sample (340) were made up of people who either had access only to a regular bike, or did not engage in any cycling activity at all. Most participants were from the Oslo region. They responded to a questionnaire in May/June 2014 (T0), prior to buying an e-bike (henceforth referred to as the *customers* group), and a follow-up (T1) four to 6 weeks later (45 had then bought an e-bike).

In addition, a sample of 28 people both physically inactive and physically active were recruited through a Norwegian NGO (*Framtiden i vaare hender*), where they were part of a project entitled “*E-bikes for a mobile life*” (henceforth referred to as the *FIVH* group). Approximately 2/3 of the participants had an inactive lifestyle, and the rest were classified as being active. The participants received a subvention for the e-bike and they were followed for a period of 6 months. A representative from the NGO contacted the intervention group and had three meetings throughout the period. No specific training was given to the intervention provider. The participants were from Oslo and a city far north in Norway (Tromsø). They responded to a questionnaire prior to the intervention (using an e-bike), and a follow up four to 6 weeks later (where 20 participants responded to the questionnaire).

A comparison group (with no e-bike) was recruited through the National Register of Bicycle Owners (a voluntary register for reducing ‘own risk’ in insurance cases).^2^ 1995 responded to the questionnaire at baseline (T0) and 765 on the follow-up questionnaire (T1). These respondents lived in the Oslo region.

### Measures

To measure *hedonism/pietism* we provided five items in the form of statements, the first the result of a pilot study in which a small group of participants were asked to think of behaviour that was most typical of hedonistic people. The other four statements were from “Norsk Monitor”, an annual survey aimed at capturing values and consumer behaviour of a representative sample of the Norwegian population (IPSOS MMI). Participants were asked to report the extent to which the statements applied to them (1 = fit poorly; 7 = fit very well). Statements 2 and 3 capture the dimension hedonism–prudence, while 4 and 5 capture Materialism–Antimaterialism.

1. I feel bad if I spend a day off doing nothing in particular.
2. It is not good for people to get everything they want.
3. In the future, I want to follow my desires and enjoy life’s pleasures.
4. I do without some material goods to live the way I want to.
5. I would rather spend money on things I can enjoy for many years than on one-off pleasures such as vacations, dining out, etc.

Perceptions about e-bikes were measured by five statements that were intended to capture Attitudes:

1. E-bikes are foolish
2. People can just as easily use an e-bike as an ordinary bike (recoded)^3^
3. E-bikes are only for people with disabilities.
4. E-bikes are no more dangerous than ordinary bikes (recoded)
5. People who buy an electric bicycle are lazy.

*Willingness to purchase* an e-bike was measured with the question: “If you were to buy a bike today, would you consider an e-bike?” Possible answers were: Don’t know; definitely not; do not think so; yes, maybe; yes, absolutely.

We measured *bicycle use* with the question: “Approximately, how far (in kilometres) did you ride your bike in the past week, i.e. in the past seven days?” Respondents were to distinguish between cycling to work/school or other transportation objectives and cycling for exercise.

We measured *physical activity* from three statements based on the short version of *International Physical Activity Questionnaire* (IPAQ) [8]. First, we asked respondents to indicate approximately how many minutes they had walked in total: “How often have you walked for more than 20 minutes during the past week?” They were to include walking for transport and walking as recreation. We also asked: “How many hours during the past seven days have you spent in total on different forms of physical activity?” They were to specify between moderate and vigorous physical activity (giving a description of activities) and only to include activities of 10 or more minutes’ duration and exclude activities they had previously mentioned (i.e. walking for more than 20 min, cycling as commuting and for exercise). The questions and unit of measurement are given in Table 1.

**Table 1:** Questionaire items and unit of measurement

What	How	Original unit of measurement	Recoded measurement
Bicycle use [transport]	Approximately, how far (in kilometres) did you ride your bike for transportation during the past week?	Kilometres	Minutes
Bicycle use [exercise]	Approximately, how far (in kilometres) did you ride your bike on exercise during the past week?	Kilometres	Minutes
Physical activity (walking)	How often have you walked (as both transportation and recreation) for more than 20 min during the past week?	Number of times	Minutes
Moderate physical activity (IPAQ)	How many hours during the past 7 days have you spent in total on moderate physical activity?	Hours	Minutes
Vigorous physical activity (IPAQ)	How many hours during the past 7 days have you spent in total on vigorous physical activity?	Hours	Minutes

### Analysis

The data were analysed using SPSS Statistics 22. A multivariate model was formulated using structural equation modelling (AMOS 22). There are two components in a structural equation model – the measurement model and the structural model. The measurement model describes relations between measured and latent variables, and can be compared to what is done in a traditional factor analysis. The structural model is the relationship between observed variables. Since we assume that Hedonism can have an effect both on the interest in buying an e-bike and on existing cycling and physical activity levels, we are interested in looking at mediating effects. Use of a structural model allows both indirect and direct effects to be estimated. Thus, SEM can carry out factor analysis, multiple regression analysis and path analysis simultaneously, and can provide a much more flexible and intuitive approach to mediation analysis than traditional regression models.

Owing to missing values, the sample size of the model is lower (*N* = 1953) than the total sample (*N* = 1995). Although there are a number of ways by which to assess model fit for structural models, the most common is the simple probability level (*p)*, but this can be misleading with large samples [26]. The goodness-of-fit index (GFI) and the adjusted root mean square error of approximation (RMSEA) are often used as alternatives. In cases such as ours, with as many as 1953 respondents, another approach is to look at the chi square/degree of freedom ratio, also known as the relative chi square [25]. A rule of thumb is that the chi square should be less than two times its degrees of freedom.

For comparison of measurements related to physical activity and cycling, we recoded bicycle use, walking frequency and physical activity into minutes. According to Ainsworth et al. [1], moderate physical activity includes cycling with light effort (10–12 mph/16–19 km/h), while vigorous physical activity is cycling fast (14–16 mph/22–25 km/h). Function and recoded unit measurements are presented in Table 2. For comparing between the groups, we used one-way Analysis of Variance (ANOVA).

**Table 2:** Function and recoded unit measurement

What	Function	Recoded unit measurement
Bicycle use for transport purposes^a^	3.39 x kilometres	Minutes
Bicycle use for exercise^b^	2.49 x kilometres	Minutes
Physical activity (walking)	Number of times × 20 min	Minutes
Physical activity moderate (IPAQ)	Hours × 60 min	Minutes
Physical activity vigorous (IPAQ)	Hours × 60 min	Minutes

^a^(16.09 + 19.31)/2 = 17.70 km/h. 60 min/17.70 = 3.39 min per km

^b^(22.53 + 25.75)/2 = 24.14 km/h. 60 min/24.14 = 2.49 min per km

## Results

### Population characteristics

Background variables (reported at baseline) for customers, *FIVH*, the comparison group and the total sample are presented in Table 3.

**Table 3:** Background variables for the different samples. N (Per cent)

	Customers		FIVH		Comparison		Total sample comparison group at baseline	
Female	26	(58%)	11	(52%)	264	(34%)	818	(41%)
Employed	37	(82%)	15	(71%)	672	(88%)	1695	(85%)
Cycle more than 4 days a week for transport	12	(27%)	1	(5%)	278	(36%)	378	(34%)
Cycle more than 4 days a week for exercise	4	(9%)	0	(0%)	59	(8%)	159	(8%)
Access to a bicycle	41	(91%)	18	(86%)	752	(98%)	1955	(98%)
Mean age	44.2		45.4		46.2		44.8	
N	45		21		767		1995	

Females are overrepresented among customers and FIVH participants (58 and 52%) compared to the comparison group (34%). In the FIVH group, only 5 % reported that they cycle regularly for transport (more than 4 days a week), compared with 27% in the customer group and 36% in comparison group. Also, the amount of exercise cycling differed among groups, from zero in the FIVH group to 36% in the comparison group. The employment rate was lower (71%) in the FIVH group than in the other groups (82 and 88%, respectively). All reported differences between groups were statistically significant (*p* < 0.01).^4^ The bicycle ownership rate was significantly different among the groups, even if most of the participants ±90%) did have access to a bike. There was no significant age difference among the groups.

### Factor analysis

Data from all three samples were used for factor analysis and subsequent SEM models.

All five items pertaining to Hedonism and Materialism were subject to principal components analysis. The variables were not highly correlated, and the Kaiser-Meyer-Oklin value was 0.48, just below the recommended value of 0.5 [6]. Interpretation of the factors therefore had to be made with some caution. Analysis revealed the presence of two components with eigen values above 1, which explained 26 and 24% of the variance, respectively. Varimax rotation, performed to aid interpretation of these components, revealed a simple structure, and non of the items strongly loaded on more than one component (Table 4). The two-component solution explained a total of 50% of the variance. The first was interpreted as covering the latent construct “Hedonism” and the second as covering “Materialism”. This was as expected. However, contrary to original interpretation, item 5 belongs to the Hedonism construct and 3 to the materialism construct. On this basis, we decided to use items 1, 2 and 5 for further analysis.

**Table 4:** Factor analysis. Extraction Method: Principal Component Analysis. Rotation Method: Varimax with Kaiser Normalization

Item		Component	
		1 “Hedonism”	2 “Materialism”
1	I feel bad if I spend a day off doing nothing special	0.667	−0.068
2	It is not good for people to get everything they want	0.709	0.24
3	In future, I want to follow my desires and enjoy life’s pleasures	−0.069	0.663
4	I forgo some material goods in order to live the way I want	0.017	0.814
5	I would rather spend money on things I can enjoy for many years than one-off pleasures such as vacation, dining out, etc.	0.559	−0.269

### SEM analysis

#### Measurement models

Measurement models were initially tested for each of the three latent variables – Hedonism, Attitudes and Physical Activity. Hedonism was derived from three items. Attitudes to e-bikes was constructed from five items. Physical activity was constructed from the two physical activity variables, as well as number of minutes spent cycling for exercise. Physical activity variables were Z-standardized, as these were heavily skewed (high number of 0 s). The two cycling activity variables were also skewed (35 and 45% reported zero minutes for transport and exercise respectively), but were kept non-transformed, as Z-transformation did not improve skewness. Due to the large number of zero values, categorising these two variables would not have improved skewness either. To assess model-data fit, we used cmin/df, (should be lower than 2) and RMSEA (should be .08 or less).

Measurement models for Hedonism and Physical Activity were both saturated, and goodness of fit tests are not applicable. The measurement model for Attitudes achieved good model fit values (cmin/df = 1.4, *p* = 0.00. RMSEA = 0.02). All measurement model parameters are presented in Figure S1 (Additional file 2: Figure S1). Cronbach’s alpha coefficients for the construct Attitudes was .68. The constructs Physical activity and Hedonism consisted of only three items each and reliability and internal consistency was assessed by use of the SEM measurement models which in such cases provide a more substantive insight into the assumed construct than alpha [19], see next section for this analysis.

#### Model development

**Fig. 1:**
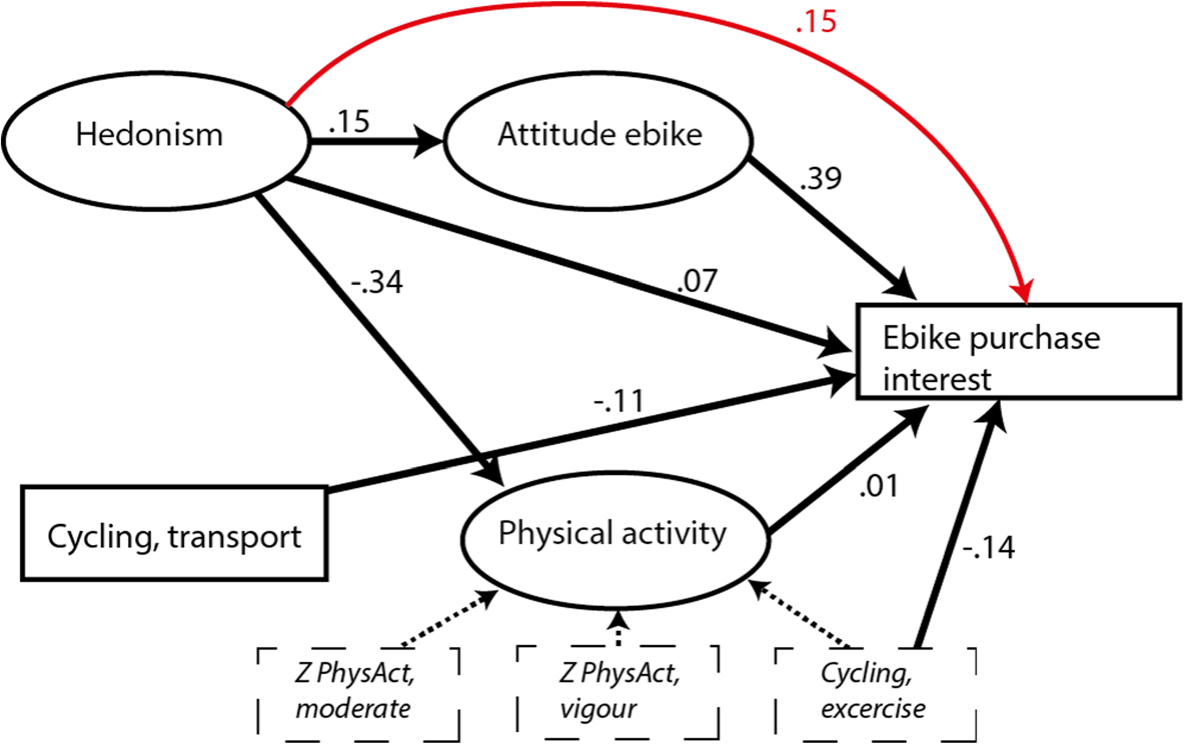
Summary of SEM model. Standardized direct effects of latent variables and non-latent (directly measured) variables. Total effects in red

To test the joint effect of the latent variables “Hedonism” and “Attitudes” to e-bikes on people’s interest in purchasing an e-bike and in their physical activity, a structural equation model (using full-information maximum likelihood methods for model estimation) was formulated using AMOS (Fig. 1). The model aims to capture both the latent structure of the independent variables as well as the causal paths from the values via attitudes/physical activity to purchase interest. Cycling activity for transport was included as an independent variable.

The model showed an acceptable fit to data (cmin/df = 1.9, *p* = 0.00. RMSEA = 0.02). Modification indices were used to improve the model on the most relevant paths, but some suggested paths were not included as they did not substantially contribute to the research questions. Including them would have improved the model fit, and would thus have rendered the *p*-value significant (as it should ideally be), but would not have altered the main conclusions below.

The model violated assumptions of multivariate normality, which is not uncommon for survey data. A common procedure to test the robustness of SEM models is to use parametric bootstrapping [5]. Therefore, the maximum likelihood models were compared with the output of bootstrapping of maximum likelihood estimates in order to evaluate potential bias (2000 bootstrap samples). For the bootstrapping analysis, overall fit of the models was calculated using the Bollen-Stine bootstrap approach in place of the chi-square statistic [5]. We found that the model was not robust, since the obtained chi-square value ranked lower than all but two out the 2000 bootstrap sampled values (Bollen-Stine *p* = .001). One important contributor to non-normal distribution in our model is the large portion of respondents reporting zero cycling or physical activity. A model was therefore tested where those who reported zero cycling for transport were excluded. This model (*N* = 1244) achieved better overall model fit than the original model (Bollen-Stine *p* = .12, cmin/df = 1.4, *p* = 0.00. RMSEA = 0.02). However, inspection of the parameter estimates showed that they did not deviate substantially from those of the original model. Since people who do not use their bicycle much is of particular relevance to the e-bike as a physical activity intervention, we decided to keep the (inferior) model with all respondents.

#### Structural equation model

Figure 1 is a summary of the *direct* effects between the most important variables in the model, i.e. all the latent variables and cycling for transport (km’s), and interest in purchasing an e-bike. All covariates and the measurement variables for the latent variables “Hedonism” and “Attitude to e-bikes” were omitted for ease of interpretation. Measurement variables for the latent variable physical activity were included. Standardized *total* effects (direct and indirect) of the structural model are summarized in Table S1 (Additional file 1: Table S1), and the complete model with all covariates and error terms is found in Figure S1 (Additional file 2: Figure S1).

There was a substantial negative contribution from Hedonism to physical activity (−0.34), and a smaller contribution to Attitudes to e-bikes (0.15) and to E-bike purchase interest (0.07). However, the total effect from Hedonism to E-bike purchase interest was greater (0.15) than the direct effect due to the indirect effect via attitudes shown in red in the figure.

Physical activity did not explain interest in purchasing an e-bike except for the item cycling for exercise (−0.14). Attitudes explain interest in purchasing an e-bike (0.39). Cycling for transport was negatively associated with interest in the purchase of an e-bike (−0.11).

### Intervention data

#### Total bicycle use (minutes)

To explore the changes in cycling activity, we recoded kilometres cycled as minutes. The results for each group at T0 and T1 (post intervention) are presented in Table 5.

**Table 5:** Changes in cycling activity per week for all groups. Minutes

	Customers		FIVH		Comparison	
	T0	T1	T0	T1	T0	T1
Exercise	38.9	54.0	11.7	34.7	54.0	44.0
Transport	63.3	172.7	12.5	200.0	93.0	85.8
Total	102.2	226.6	24.2	234.7	146.9	129.8
N	45	45	20	20	757	757

For customers and the *FIVH* group, the total cycling activity (exercise and transportation objectives) increased from T0 to T1 (with 124.4 min and 210.5 min, respectively). In the comparison group (−17.1 min), there was a decrease in cycling activity (non-significant).

#### Changes in physical activity

To explore the effect on other physical activity followed by changes in cycling activity, we looked at minutes reported for walking, moderate physical activity (MPA) and vigorous physical activity (VPA). The results for each group are presented in Table 6.

**Table 6:** Minutes of physical activity per week in all groups at T0 and T1

	Customers		FIVH		Comparison	
	T0	T1	T0	T1	T0	T1
MPA	149.3	164.0	135.0	225.0	161.1	165.1
VPA	70.7	86.7	21.0	87.0	104.3	112.8
Walking	88.0	76.9	74.0	61.0	81.5	76.0
Total	308.0	327.6	230.0	373.0	346.9	353.9
N	45	45	20	20	757	757

For all three groups, the total physical activity (walking, MPA, VPA) of e-bike users increased from T0 to T1, but only for the *FIVH* group were the changes significant (143.0 min, *p* < 0.000).

#### Changes in overall physical activity

In exploring the changes in overall physical activity, we aggregated values for total MPA (cycling for transport, walking activity and other MPA) and total VPA (cycling for exercise and other VPA).

**Fig. 2:**
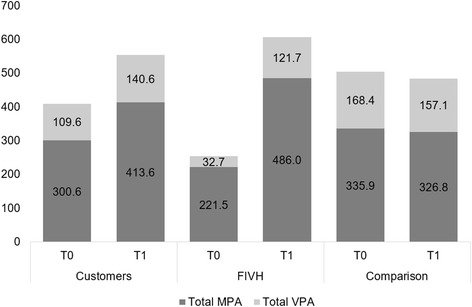
Changes in overall physical activity of all groups. Minutes

The results presented in Fig. 2 illustrate the changes in overall physical activity for the different groups – changes in total physical activity for both customers and the *FIVH*. To test whether they are significant, one-way Analysis of Variance (ANOVA) was performed for changes in MPA, VPA and total physical activity, respectively.

The changes in overall physical activity were significant for the e-bike users (143.9 min, F = 7.515, *p* = 0.006) and for the *FIVH* group (353.5 min, F = 17.689, *p* > 0.000) compared to the comparison group (−20.4 min). In the e-bike group, only the changes in MPA were significant (112.9 min, F = 7324, *p* = 0.007). In the *FIVH* group, changes in both MPA (264.5 min, F = 16.464, p > 0.000) and VPA (89.0 min, F = 3.41, *p* = 0.06) were significant. On comparing the two different groups of e-bike users, the changes were greater for *FIVH* than for customers (F = 6.298, *p* = 0.015) for the MPA (F = 4.435, *p* = 0.039) and VPA (F = 2.863, *p* = 0.96) looked at separately.

## Discussion

The SEM model suggests that people who score high on Hedonism are more interested in buying an e-bike and are less physically active than those who score low on Hedonism. People who cycle a lot, both for transport and for exercise, are less interested in buying an e-bike. The results show that e-bike interest is not directly linked to existing physical activity, but that it could be explained via scores on Hedonism. Hence, the results point potentially to a positive health effect, since the e-bike seems to have a stronger appeal among those who have a lower level of physical activity, so-called “couch potatoes”.

The use of the latent construct Hedonism in the SEM model deserves some discussion. Hedonism was included in order to capture what we believed to be a potentially important intrinsic motivation for travel [31] of particular relevance to physical activity and the electric bicycle. The measurement model for this construct was saturated and the psychometric properties of our construct needs to be assessed in relation to the full model. In the full model, all Hedonism items had satisfactory parameter estimate sizes (standardised: 0.3–0.4), and the full model showed acceptable model fit. One previous study has compared the IPSOS/MMI items utilised here with the internationally validated Portrait Value Questionnaire (PVQ) [36], and found that they discriminate similarly across different segments of the population [40], which indicates a certain level of construct validity. However, we cannot be sure that we have really captured well the purported essence (that is of most value) of Hedonism. Future studies should therefore consider using validated questionnaire items such as those from the PVQ in order to verify our results.

It should also be noted that the cycling activity and physical activity variables used in the model were heavily skewed. Excluding respondents who did not cycle or exercise at all would have improved model fit, and was considered but rejected, since these respondents are of particular relevance for the e-bike as an activity generating tool. The bootstrap tests revealed that the inclusion of the skewed variables did not affect the results substantially.

The study then went on to explore the health effect of increased cycling, with the introduction of e-bikes in “real life settings” in light of physical activity and substitution. The results showed that cycling activity (both for transport and for exercise) increased in the two groups of e-bike users while remaining stable (non-significant decrease) in the comparison group. We found that increased cycling for transport (moderate physical activity) leads to more total physical activity in the group of e-bike users compared to a comparison group. The changes for both cycling activity and other physical activity were greatest for those in the *FIVH* group. Hence, our findings indicate that there is no substantial substitution effect for physical activity with the introduction of e-bikes in a Norwegian cycle population.

For the intervention study, participants were recruited from two different samples – e-bike customers and a selected group of people with marked sedentary behaviour (the *FIVH* group). For those in the *FIVH* group, the potential for change in both cycling activity and other physical activity was greater than for the other groups, which might explain the dramatic change in cycling activity. Close follow-up of this group included introduction of the use of the bicycle and three meetings throughout the trial period, may also have contributed to the dramatic increase. In light of this, we needed to address this group as a sub-group of e-bike users, and not representative of the total population of e-bike users. Still, these dramatic results illustrate the potential of the e-bike to induce increased physical activity among those with a sedentary lifestyle who might find a regular bicycle as a non-alternative.

Looking at the group of *customers* gives a more representative picture. Our results showed that for those who already had moderate cycling activity at baseline (with a regular bike), the increase in cycling activity was significantly higher than for those in the comparison group. The e-bike users replicate their previous cycling activity with a regular bike, but the duration of the activity (numbers of minutes) increases significantly. These results are consistent with previous studies [43] showing that those who purchase an e-bike cycle more often (frequency) and for longer distances (duration) than before.

There were baseline differences between the test groups and the comparison group. The test groups cycled less, had lower employment levels and more females than the comparison group. They also had a somewhat lower bicycle ownership. From previous research we know that e-bikes tend to have a larger appeal (in the form a stated preference) for those who cycle less, and for females [15]. The observed baseline differences (which are the results of an actual purchase decision) functions to confirm these findings.

It should be noted that for all groups the reported levels of PA at T0 were well above recommendations. This might seem to contrast with the results of the SEM analysis, showing that e-bikes seems to appeal to the most sedentary population (one would maybe expect the FIVH group and the customer group to have lower than recommended activity levels). There are two likely explanations for this discrepancy. First, the levels of physical activity could have been systematically overrated, either due to self-report bias, or due to the way in which we calculated the level of PA from the kilometres cycled (into minutes). This explanation is partly supported by the fact that the FIVH group were also recorded as having above recommended values of physical activity, even though this group had been carefully screened in the selection process. Another likely explanation is that the SEM analysis investigates peoples *stated* preferences for e-bikes, whereas the intervention study looks at the results of *revealed* preferences (i.e. having bought an e-bike). In other words, those who end up buying an e-bike might have higher activity levels than those who express an interest for one. Even if both explanations are true, our initial conclusion, that the e-bike appeals to “couch potatoes” is still valid, since those who bought an e-bike had lower activity levels than the comparison group at baseline.

### Limitations and strengths

A strength of the current study is the fairly large sample size.With certain limitations, it is also representative of the cycling population of Norway. The study does not aim to capture the views and attitudes of the population as a whole, but of people who already own a bicycle and thus are more likely to be interested in purchasing an e-bike.

A further strength is the design, (i.e. prospective repeated measures design with test and comparison groups) A challenge with any study of cycling activity in Norway is the large seasonal variation in cycling. Since the current study is conducted in the same time period as this natural seasonal variation, we need to take this into account when assessing the effect of the e-bike on cycling activity. To do this properly we used a comparison group that was intended to resemble the test group as much as possible, but that was not provided with an e-bike. Hence, we assumed that the largest single change over time was the introduction of an e-bike.

There were baseline differences between the samples. The differences in cycling levels should not be detrimental to the observed effects of an e-bike. The changes in physical activity levels will be lower if people who already cycle much were to obtain an e-bike. There was a small difference in the rate of bicycle ownership. Thus, it could be argued that it was the effect of gaining access to a bicycle of any kind, and not to an e-bike per se, that influenced people’s activity levels. However, even in the group with the lowest ownership (the FIVH group) 18 out 21 participants owned a bicycle. It is unlikely that the dramatic effect that was observed could be isolated to the last three participants.

A potential limitation of the study is the use of self-reported measurements in the form of questionnaire items. Within the field of transport research, travel data are traditionally measured by surveys. A common criticism of these surveys is that people tend to under-report journeys related to walking and cycling, and the reported distances (kilometres and time) are not precise [20]. The distribution of smart phones and the development within app technology imply great potential for collecting both electronic travel data and physical activity data. An aim of future studies should be to include more objective measures for comparison and for validating self-report measures.

The studywas performed in Oslo, Norway where the e-bike market is still quite immature, and should be interpreted with this in mind. First, we studied an urban setting, and our results might not necessarily apply to more rural areas, where limited cycling infrastructure and long travel distances might deter uptake of cycling (even with an e-bike). Second, demographics and travel patterns differ between countries. It is likely that similar results would be obtained in countries with similar characteristics, most notably with similar cycling levels. However, it is not certain whether these results can be replicated in countries where the e-bike already has gained a strong market position.

Another aspect is the short follow up period (4–6 weeks), which is maybe too short to determine sustained behaviour change. Future studies should aim to explore if the changes in PA would remain for a prolonged period.

Although the baseline sample is large, our intervention group counts only 65 persons, which is a limitation. On the other side, the number is not small for an intervention design the physical activity effects are substantial enough to be significant even with this number of participants.

Our measure of physical activity was inspired by the short version of *International Physical Activity Questionnaire* (IPAQ) [8]. In the validated IPAQ questionnaire the respondents account for how many days they have conducted different types of activities, and approximately how many hours and minutes they “normally” used on one of these days [8]. In our study, we wanted a more detailed report to account for the differences in the activity within 1 week, and the respondents reported how many hours in total they had been physically active during the past 7 days. By doing so, we reduce the challenge of averaging quite infrequent activities for the respondent (what is the “normal duration” of moderate physical activity for a person who has cycled for 20 min on Monday and played football for 90 min on Thursday?)

For walking, a cut-off value 20 min was introduced, to make it easier to remember and hence report. The latter might have contributed to some physical activities not being accounted for and resulting in an *underreporting* of physical activity. Since the results showed no reduction in physical activity, we argue for the absence of a substitution effect followed by increased cycling activity.

It can be discussed whether the short version of IPAQ is sensitive enough to answer the research questions. The categories are broader than in the long version, and it is possible that those who used e-bikes might have overestimated their activity. From a training-oriented perspective, the latter would be a bigger problem. From a public health perspective (where the threshold is moderate activity), we argue for the measurement of physical activity used in this study to be sensitive enough. Still, a limitation in our study is that we did not address the amount of sedentary behaviour. This would have been an important measurement from a public health perspective, as prolonged time spent in uninterrupted sedentary behaviour can provide health risks independently regardless of engagement in physical exercise [13, 30].

We have explored the health effect of cycling on an e-bike by measuring the amount of time spent on the bicycle. The health effect of physical activity is not merely related to the duration of the activity, but also to the individual’s baseline fitness (i.e. weight, intensity, etc.) [4]. Since these factors were not accounted for in this study, we cannot clearly express the health effect of each individual, but instead estimate the total effect with a public health perspective – which was the aim of our study. Future studies should aim at providing baseline and follow-up measurements of objective fitness to arrive at more precise results, and also potentially to differentiate between different user groups.

### Implications

From a theoretical point of view, it could be argued that the construct we have called Hedonism is rather a measure of what is known as self-regulation or self-control in motivational theory. A distinction can be made between maladaptive Hedonism and value-based (Schwarz type) Hedonism [27], where the former can be seen as akin to but still different from the personality trait self-control. In the current study, the items used were borrowed from a survey battery that only loosely relates to Schwarz’s theories about altruistic versus hedonistic values. Still, their substantive meaning was closer to a value-oriented understanding (“for me, it is important to …”) of concept, than to a personality-oriented understanding (“I am a person who …”). The value approach is usually studied in conjunction with environmental behaviour, while the personality approach is typically related to health behaviour. This therefore raises the interesting question whether e-bike purchase is health behaviour or environmental behaviour. Previous research on consumer behaviour for environmental technology has found little or only mixed evidence of environmental motives behind the purchase of, for example, electric cars [44]. Another study, one looking at motives for e-bike purchase, found no effect of environmental values on purchase interest [16]. These results, seen in conjunction, point to e-bike purchase as being motivated more by health concerns than by environmental concerns.

In Norway, there has been discussion about governmental help for e-bike purchase, support in the form of fiscal incentives such as Value-added-tax (VAT) exemptions. Normally, the debate is based on environmental objectives (i.e reduced local and global pollution), but based on these findings we argue that it is possible to support such initiatives just as much with public health in mind, at least in countries were uptake of e-bikes, or cycling levels still are low.

## Conclusion

The current study looks at the potential public health effects of e-bikes and explores the changes in physical activity of users. While e-bike users increase the amount of cycling they do, as well as the levels of both moderate and vigorous physical activity, no such difference is found in the comparison group. The results show that with increased cycling for transport more total physical activity is accumulated in the e-bike user group compared to a comparison group, hence our findings indicate that there is no substitution effect of physical activity with the introduction of e-bikes in a Norwegian cycle population. The study also looks at perceptions in relation to e-bikes and physical activity among the general public, and shows that the appeal of the e-bike is strongest among those with little existing interest in, or levels of, physical activity. The net effect of the e-bike therefore seems positive from a public health perspective.

## Additional files

Additional file 1: Table S1. Standardized *total* effects (direct and indirect) of the structural model. (XLSX 8 kb)

Additional file 2: Figure S1. Complete model with all covariates and error terms. (PNG 147 kb)

## Abbreviations

ANOVA: One-way analysis of variance
FIVH: Framtiden i vaare hender (eng: The future in our hands)
IPAQ: International Physical Activity Questionnaire
MPA: Moderate physical activity
SEM: Structural equation modelling
VAT: Value-added-tax
VPA: Vigorous physical activity

## Footnotes

1 MET is a physiological measure expressing the energy cost of physical activities and is defined as the ratio of metabolic rate (rate of energy consumption) during a specific physical activity to a reference metabolic rate. 1 kcal/kg/time = 1 MET

2 Approval for use of third party data (e-mail)

3 Number 2 and 4 were recoded for the analysis (1 = 7 etc.)

4 Pearson Chi-Square, *p* < .05
