# Supplementary figures and images for: A push for public health: the effect of e-bikes on physical activity levels
Source: BMC Public Health. 2017 Oct 16;17:809. doi: 10.1186/s12889-017-4817-3 (PMC5644161; doi:10.1186/s12889-017-4817-3)

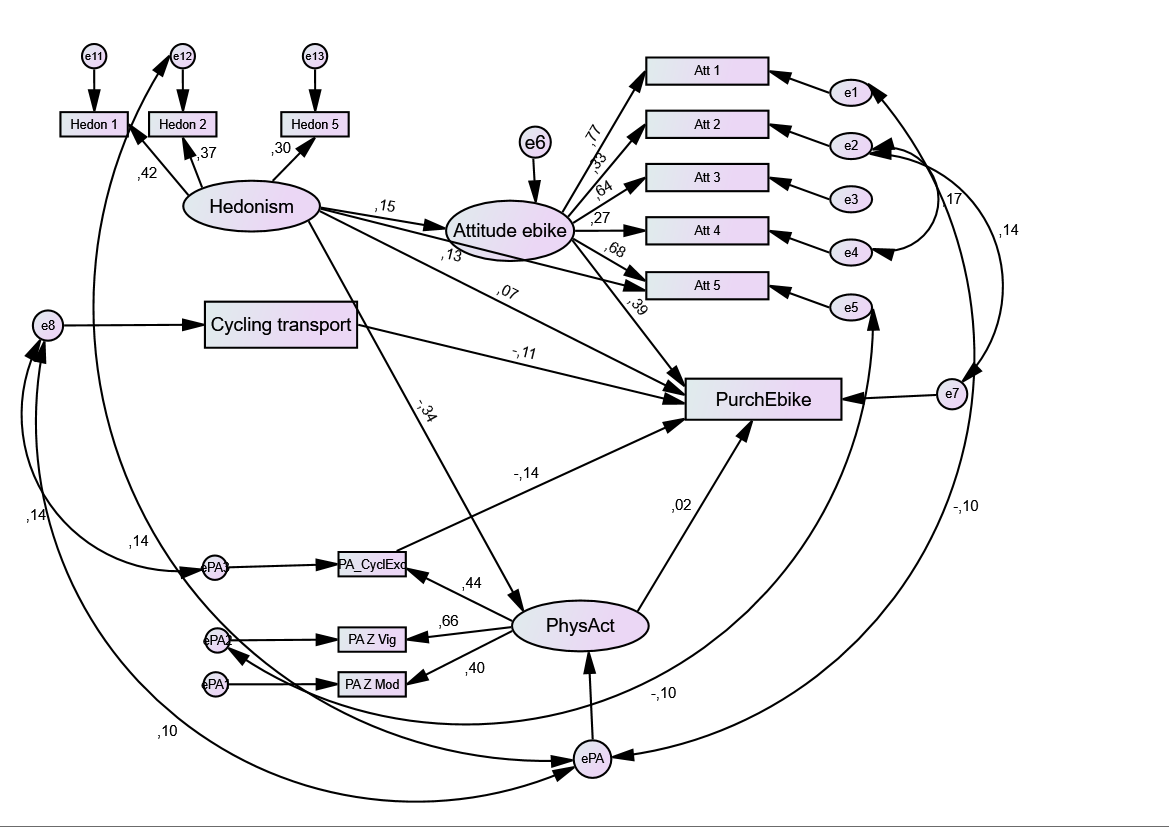

Supplement: Supplementary file 2 — Complete model with all covariates and error terms. (PNG 147 kb) [file 12889_2017_4817_MOESM2_ESM.png]
